# *Ex vivo* Expansion of Bovine Corneal Endothelial Cells in Xeno-Free Medium Supplemented with Platelet Releasate

**DOI:** 10.1371/journal.pone.0099145

**Published:** 2014-06-19

**Authors:** Ming-Li Chou, Thierry Burnouf, Tsung-Jen Wang

**Affiliations:** 1 Graduate Institute of Medical Sciences, College of Medicine, Taipei Medical University, Taipei, Taiwan, R.O.C.; 2 Graduate Institute of Biomedical Materials and Tissue Engineering, Taipei Medical University, Taipei, Taiwan, R.O.C.; 3 Department of Ophthalmology, Taipei Medical University Hospital, Taipei, Taiwan, R.O.C.; Department of Ophthalmology, School of Medicine, College of Medicine, Taipei Medical University, Taipei, Taiwan, R.O.C.; National Taiwan University College of Medicine, Taipei, Taiwan, R.O.C.; and Department of Ophthalmology, National Taiwan University Hospital, Taipei, Taiwan, R.O.C.; Rutgers - New Jersey Medical School, United States of America

## Abstract

Clinical-grade *ex vivo* expansion of corneal endothelial cells can increase the availability of corneal tissues for transplantation and treatment of corneal blindness. However, these cells have very limited proliferative capacity. Successful propagation has required so far to use very complex growth media supplemented with fetal bovine serum and other xenocomponents. We hypothesized that human platelet releasates rich in multiple growth factors, and in particular neurotrophins, could potentially be a useful supplement for *ex vivo* expansion of corneal endothelium cells due to their neural crest origin. Platelet releasates were prepared by calcium salt activation of apheresis platelet concentrates, subjected or not to complement inactivation by heat treatment at 56°C for 30 minutes. Platelet releasates were characterized for their content in proteins and were found to contain high amount of growth factors including platelet-derived growth factor-AB (30.56 to 39.08 ng/ml) and brain-derived neurotrophic factor (30.57 to 37.11 ng/ml) neurotrophins. We compared the growth and viability of corneal endothelium cells in DMEM-F12 medium supplemented with different combinations of components, including 2.5%∼10% of the platelet releasates. Corneal endothelium cells expanded in platelet releasates exhibited good adhesion and a typical hexagonal morphology. Their growth and viability were enhanced when using the complement-inactivated platelet releasate at a concentration of 10%. Immunostaining and Western blots showed that CECs maintained the expressions of four important membrane markers: Na-K ATPase α1, zona occludens-1, phospho-connexin 43 and N-cadherin. In conclusion, our study provides the first proof-of-concept that human platelet releasates can be used for *ex* *vivo* expansion of corneal endothelium cells. These findings open a new paradigm for *ex* *vivo* propagation protocols of corneal endothelium cells in compliance with good tissue culture practices and regulatory recommendations to limit the use of xenogenic materials.

## Introduction

The corneal endothelium, an essential part of the cornea, is composed of a unique thin, fragile monolayer of hexagonal cells that dwell in the innermost layer of the cornea. Corneal endothelial cells (CECs) are embryologically derived from the neural crest. They cover the posterior surface of Descemet’s membrane and make contact with the aqueous humor [1]. CECs constitute a physiological and tight intercellular barrier that pumps fluids across the cornea, regulating hydration of the corneal stroma [2], and contributes to maintaining transparency and clarity for optimal visual functions [1], [3]–[6]. Damage, injury, and pathologies, like intraocular surgery and Fuchs dystrophy, cause CECs to crumble, lead to excessive hydration and opacification of the cornea, and result in severe visual impairment. Repairing CECs is very challenging since these cells exhibit a very limited proliferative capacity [3]. Corneal transplantation, which is needed by an increasing number of blind patients worldwide due to aging populations, is the only current option for visual restoration with corneal blindness [7], [8]. The critical shortage of adequate tissue quality limits the availability of donor corneas [9], supporting the development of *ex* *vivo* CEC expansion to improve the supply for clinical-grade transplantation.

CECs have long been thought to be unable to expand *ex* *vivo*. However, expansion of CECs was achieved using complex culture medium supplemented with fetal bovine serum (FBS), purified xenogenic proteins, bovine insulin, recombinant growth factors [such as epithelial growth factor (EGF) and platelet-derived growth factor (PDGF)-BB], pituitary extracts, and others compounds like cholera toxin [4], [10]–[15]. Nevertheless, such materials have drawbacks, including, for instance the case of components of bovine origin, exposing recipients to immunological [16], [17], viral [18], and/or prion [19] risks. As such, they are discouraged or prohibited by regulatory authorities and the World Health Organization [20], [21]. In addition, many recombinant growth factor supplements are either only of reagent-grade quality, not licensed for this specific application, or very expensive. Alternative xeno-free supplements for CEC expansion therefore need to be identified. Considering the neural crest origin of the CECs, we hypothesized that human platelet releasates, which contain a complex mixture of growth factors, including PDGF, vascular endothelial growth factor (VEGF), and brain-derived growth factor (BDNF) neurotrophins [22], could potentially be used as a sole supplement of basal growth medium for *ex* *vivo* expansion of CECs. To our knowledge, this is the first study reporting the use of human platelet releasates as a growth medium supplement to isolate and expand primary CECs.

## Materials and Methods

### Collection of Platelet Concentrates

**Figure 1:**
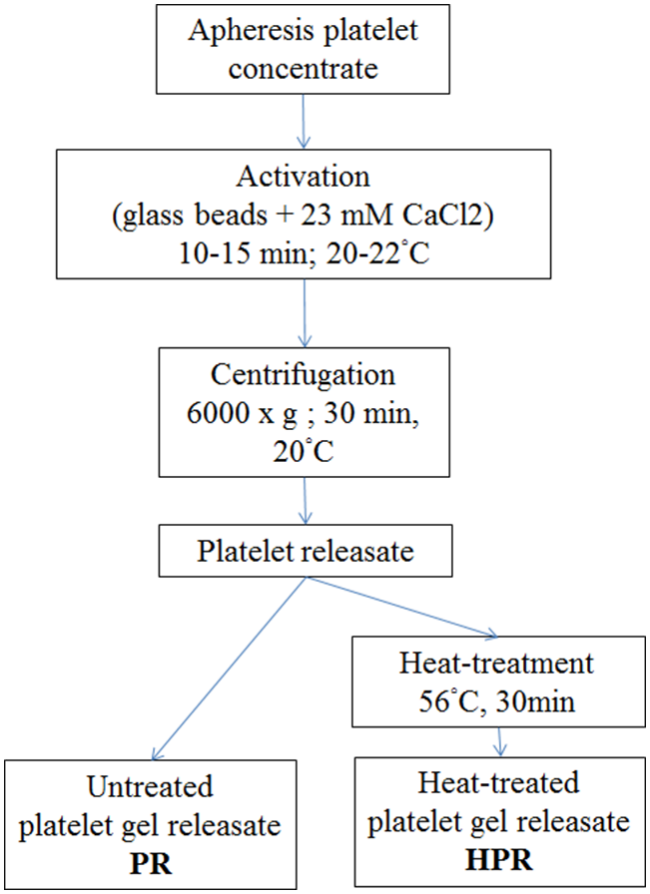
Preparation of platelet materials. Platelet releasate (PR) and heat-treated PR (HPR) were obtained by 23 mM calcium chloride activation for 10∼15 min at 20∼22°C in the presence of glass beads. The fibrin-free supernatant was clarified at 6000×*g* for 30 min at 20°C. Half of the releasate (PR) was aliquoted and frozen at <−20°C until being used. HPR was complement-inactivated at 56°C for 30 min and frozen at <−20°C.

Platelet concentrates were prepared using a Haemonetics MCS+ apheresis machine (Haemonetics Corp., Braintree, MA, USA) from 4 volunteer donors who provided written informed consent, with approval by the Institutional Review Board of Taipei Medical University: N° 201210007. Platelet concentrates (about 280 mL each) were stored at room temperature under mild mixing on a platelet agitator at 22±2°C for 1∼3 days after collection, and were individually processed as described below and summarized in Fig. 1. The platelet concentrate (1 ml) was taken aseptically under laminar flow to determine the blood cell count and hemoglobin, as in our previous research, using a cell counter (ABC Vet automatic blood counter, ABX Diagnostics, Montpellier, France) [23].

### Preparation of Platelet Releasate (PR)

The preparation procedure is summarized in Fig. 1. Briefly, platelet concentrates were aseptically taken from the collection bag and individually transferred to Falcon tubes (Becton Dickinson Biosciences, Franklin Lakes, NJ, USA) containing glass beads that were previously sterilized by autoclaving, as we described before [24]. Sterile CaCl_2_ (1 M) was added under laminar flow to the platelet concentrate to a final concentration of 23 mM and mixed gently for 10∼15 min at 20∼22°C to activate the platelets and blood coagulation and convert the plasma fibrinogen into fibrin. The fibrin clot was removed by adhesion to the glass beads through vigorous shaking [24]. The fibrin-free supernatant was centrifuged at 6000×*g* for 30 min at 20°C to clarify and recover the fibrin-free supernatant. Half of the releasate (termed the PR) was aliquoted and frozen at <−20°C until being used. The other half (termed the HPR) was complement-inactivated at 56°C for 30 min in a temperature-controlled water-bath and subsequently aliquoted and frozen at <−20°C. The absence of residual blood cells in the PR and HPR was controlled before freezing by counting as indicated above. A sample of the starting PC was kept, centrifuged at 2500×*g* at room temperature to pelletize the platelet and obtain platelet-poor-plasma (PPP) that was used as a control for determining the growth factors.

### Growth Factor Determination and Protein Profile

The PR and HPR samples were thawed at 37°C and analyzed within 1 h. Growth factors were determined using the respective Quantikine enzyme-linked immunosorbent assay (ELISA) kits (R&D Systems, Minneapolis, MN, USA) following the manufacturer’s instructions, as described in our former research [25]–[27]. Standards and samples were assayed in duplicate, and mean values were calculated after taking into account the dilution factor of the samples. Sodium dodecyl sulfate-polyacrylamide gel electrophoresis (SDS-PAGE) was performed as before [24] using gradient gels, reagents, and electrophoretic systems from Invitrogen (Carlsbad, CA, USA). A prestained protein molecular weight standard (Novex Sharp, Invitrogen) was used to assess the molecular mass.

### Chemical Analysis

Contents of protein, glucose, chloride, sodium, potassium, calcium, phosphate, magnesium, iron, the total iron capacity, ferritin, vitamin B12, and folate of PR and HPR were determined using a Roche MODULE P800 Automatic Biochemical Analyzer (Roche Diagnostics, Mannheim, Germany) as previously described [27].

### CEC Isolation and Culture Conditions

Over 30 fresh bovine eyes were purchased from a local butcher, a few hours after the animals were killed at a local abattoir. The eyes were disinfected by immersion in an iodine solution for 3∼5 min and then transferred into phosphate-buffered saline (PBS; Life Technologies, Grand Island, NY, USA). CECs were isolated by peeling from the corneal endothelium sheets and digested at 37°C in 1x TrypLE Express (Gibco, Life Technologies) for 30 min, as in our previous work [11]. The medium used for isolation and culture was Supplemented Hormonal Epithelial Medium (SHEM), made of an equal volume of HEPES-buffered Dulbecco’s modified Eagle medium (DMEM) and Ham F12 (Invitrogen, Life Technologies) supplemented with 5% FBS (Gibco, Life Technologies), 5 µg/mL insulin, 5 µg/mL transferrin, 5 ng/mL selenium, 50 unit/mL penicillin, 50 µg/mL streptomycin, 250 ng/mL amphotericin B (all from Invitrogen, Life Technologies), 0.5% dimethyl sulfoxide (DMSO), 2 ng/mL recombinant human EGF (rHuEGF), and 1 nM cholera toxin (all from Sigma-Aldrich, St. Louis, MO, USA). Alternatively, the basal medium was supplemented with 2.5%, 5%, 7.5%, or 10% human platelet fractions only. After digestion, bovine CECs (BCECs) were seeded at a rate of approximately 10^4^ cells/well in a 24-well culture plate (2 cm^2^; Becton Dickinson, Franklin Lakes, NJ, USA), and cultured in an incubator (Forma 310, Thermo Scientific, Neihu, Taiwan) at 37°C in an atmosphere of 95% air and 5% CO_2_. The medium was changed every 2∼3 days. Seven combinations of cell culture conditions were evaluated (Table 1) to determine the possibility of avoiding FBS and other xenogenic or recombinant nutritional supplements (rh-EGF, transferrin, insulin, and cholera toxin), DMSO, and cholera toxin. Conditions with and without a heparin anticoagulant were also tested to prevent the risk of fibrin gel formation affecting cell cultures.

**Table 1:** Comparison of different medium conditions for in(CECs).

Condition	1	2	3	4	5	6	7
Medium Base	DMEM + F12	DMEM + F12	DMEM + F12	DMEM + F12	DMEM + F12	DMEM + F12	DMEM + F12
FBS	5%	5%	−	−	−	−	−
Human platelet lysate	−	−	5% PR	5% HPR	5% HPR	5% PR	5% HPR
7.5% Sodium Bicarbonate	+	+	+	+	+	+	+
Heparin	−	+	+	+	−	+	+
0.5% DMSO	+	+	−	−	−	+	+
2 ng/ml rHuEGF	+	+	−	−	−	−	−
5 µg/ml Insulin; +5 µg/mlTransferrin; +5 ng/ml Selenium	+	+	−	−	−	−	−
1 nM Cholera Toxin	+	+	−	−	−	−	−
1% Amp B; +1% Pen- Strep	+	+	+	+	+	+	+

DMEM, Dulbecco’s modified Eagle medium; PR, platelet releasate; HPR, heat-treated platelet lysate; rHuEGF, recombinant human epithelial growth factor.

### Morphology, Cell Count, and Cell Viability Assay

The morphology of cells was observed under a phase-contrast microscope (IX71, Olympus, Tokyo, Japan) at 1, 3, 5, and 7 days. For counting, CECs were trypsinized at 1, 3, 5, and 7 days, and viable cells were counted using a Countess Automated Cell Counter (Invitrogen) following the manufacturer’s instructions. Cell numbers were determined for triplicate wells at each time point. CECs were seeded at a rate of approximately 10^4^ cells/well into a 96-well plate (Becton Dickinson) and cultured in different media for 24 h until they reached confluence. The medium was removed and replaced with 100 µL of fresh culture medium. MTT (10 µl of a 12 mM stock solution; Invitrogen, Life Technologies) was added and incubated at 37°C for 4 h. The medium was removed, and 50 µL of DMSO was added to each well to dissolve the formazan crystals, and it was thoroughly mixed before incubation for 10 min at 37°C. The absorbance at 540 nm was measured to assess cell viability.

### Immunocytochemistry

Cells were seeded in 24-well plates and grown to confluence which typically required 3∼5 days. Cells were carefully washed with PBS and fixed in fresh 4% paraformaldehyde (PFA)/PBS at pH 7.4 for 10∼20 min at room temperature. After washing twice with PBS, PFA-fixed cells were permeabilized for 5 min in 0.2% Triton X-100 (Sigma-Aldrich) in PBS. Fixed cells were blocked in 10% BSA in PBS for 1 h at room temperature. As in our previous experiments [12], primary antibodies, including rabbit anti-Na-K ATPase α1 (1∶100 dilution; Merck Millipore, Billerica, MA, USA), rabbit anti-zona occludens-1 (ZO-1; 1∶100; Invitrogen, Life Technologies), phospho-connexin 43 (1∶100; Merck Millipore), and N-cadherin (1∶100; Becton Dickinson), were incubated overnight at 4°C. Cells were washed twice with PBS and then incubated with a 1∶1000 dilution of FITC-labeled secondary antibodies (eBioscience, San Diego, CA, USA) in blocking buffer for 1 h at room temperature. After washing three times in PBS, all cells were stained with the DAPI nuclear marker (1∶5000; Invitrogen, Life Technologies) for 20 min at room temperature. A fluorescent mounting solution was added, the fluorescence was visualized on a Nikon Eclipse E-800 fluorescence microscope (Tokyo, Japan), and images were obtained with a spot digital camera.

### Western Blot Analysis

A Western blot analysis was performed on CECs cultured in medium supplemented with FBS or HPR until they had reached confluence. In brief, cells lysis was performed using RIPA (Sigma) for 10 min on ice. Proteins from cell extracts were separated by SDS-PAGE using 4%∼12% Bis-Tris Protein Gel (Invitrogen), transferred to polyvinylidene difluoride membranes, and blocked with a blocking solution. The primary antibodies described above were used followed by appropriate secondary antibodies conjugated with alkaline phosphatase.

### Statistical Analysis

Data are reported as the mean ± standard deviation (SD). Statistical analyses of cell counts and MTT were performed using Student’s *t*-test. A *p* value of <0.05 was considered a significant difference.

## Results

### Cell Counts of Starting Platelet Concentrates

The mean platelet, white blood cell, and red blood cell counts in starting platelet concentrates were 1250±167.3×10^6^/ml, 0.85±0.078×10^6^/ml, and 0.03±0.009×10^9^/ml, respectively, typical of this apheresis platelet collection procedure. Hemoglobin was undetectable. There was no detectable blood cells in the PR or HPR.

### SDS-PAGE, Growth Factors, and Cytokines in PRs

**Figure 2:**
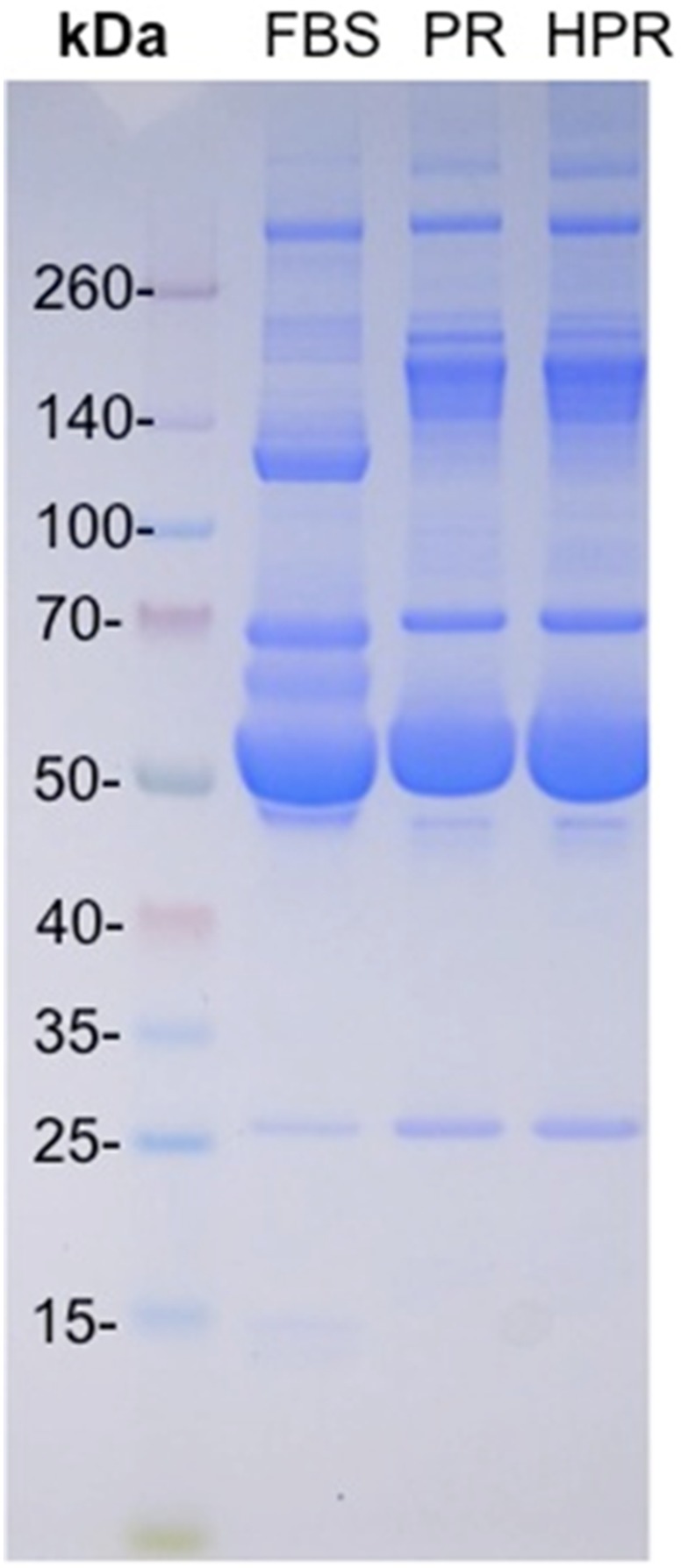
SDS-PAGE under non-reduced conditions. Molecular weight protein markers (a), fetal bovine serum (FBS, b), platelet releasate (PR, c), and heat-treated PR (HPR, d). Patterns show the absence of fibrinogen (about 260 kDa) in FBS, PR, and HPR, higher contents of immunoglobulin G (160 kDa) in the PR and HPR, and the presence of albumin (58 kDa) as the main protein component in FBS, PR, and HPR.

The SDS-PAGE protein profile of FBS, PR, and HPR (Fig. 2) showed that all three materials contained albumin (58 kDa) but had no detectable fibrinogen (260 kDa). Immunoglobulin G (IgG; 160 kDa) was present in the PR and HPR but was undetectable in FBS. The PR and HPR had protein profiles typical of human serum. The content of platelet growth factors (Table 2) was significantly higher (*p*<0.001) in the PR and HPR compared to PPP, apart from the hepatocyte growth factor (HGF). Results of the chemical analysis (Table 3) did not reveal much difference between the PR and HPR apart from less vitamin B12 in the HPR. The most important differences were more glucose, calcium, TIBC, UIBC, and ferritin, and less potassium, phosphate, iron and folate in platelet releasates compared to FBS.

**Table 2:** Contents of proteins and platelet growth factors in platelet releasate (PR) and heat-treated PR (HPR) (***p<0.001, PR and HPR, compared to platelet-poor plasma).

	Platelet-poor plasma		PR		HPR	
	Mean	SD	Mean	SD	Mean	SD
Total protein (mg/ml)	58.5	2.85	55.50	2.12	54.50	3.54
PDGF-AB (ng/ml)	2.65	0.11	39.08***	1.46	30.56***	1.22
BDNF (ng/ml)	3.51	0.15	37.11***	3.93	30.57***	0.34
VEGF (ng/ml)	ND	ND	0.21***	0.08	0.14***	0.11
EGF (ng/ml)	0.08	0.00	0.44***	0.00	0.45***	0.01
b-FGF (pg/ml)	18.61	0.00	44.79***	8.00	117.95***	4.65
TGF-β1 (ng/ml)	10.10	0.08	29.93***	1.25	33.39***	1.05
HGF (ng/ml)	0.22	0.07	0.16	0.04	0.37	0.01

PDGF, platelet-derived growth factor; BDNF, brain-derived neurotropic factor; VEGF, vascular endothelial growth factor; EGF, epithelial growth factor; FGF, fibroblast growth factor; TGF, transforming growth factor; HGF, hepatocyte growth factor; ND, not detectable.

**Table 3:** Comparative chemical analysis of fetal bovine serum (FBS), platelet releasate (PR), and heat-treated platelet releasate (HPR) (*n* = 2).

	FBS	PR	HPR
Glucose (mg/dl)	81	340	329
Chloride (mEq/l)	94	119	113
Sodium (mEq/l)	138	168	161
Potassium (mEq/l)	12.9	3.8	3.7
Calcium (mg/dl)	13.9	50.1	52.2
Phosphate (mg/dl)	10.2	3.4	3.1
Magnesium (mg/dl)	3.3	2.7	2.0
Iron (µg/dl)	195	105	104
TIBC (µg/dl)	247	945	943
UIBC (µg/dl)	52.00	840	839.00
Ferritin (ng/ml)	1.0	37	37.00
Vitamin B12 (pg/ml)	308.3	292.2	161.90
Folate (ng/ml)	7.43	1.97	2.51
Hemoglobin (g/dl)	<0.10	<0.10	<0.10

TIBC, total iron-binding capacity; UIBC, unsaturated iron-binding capacity.

### CEC Growth, Viability, and Morphology in 5% PRs

**Figure 3:**
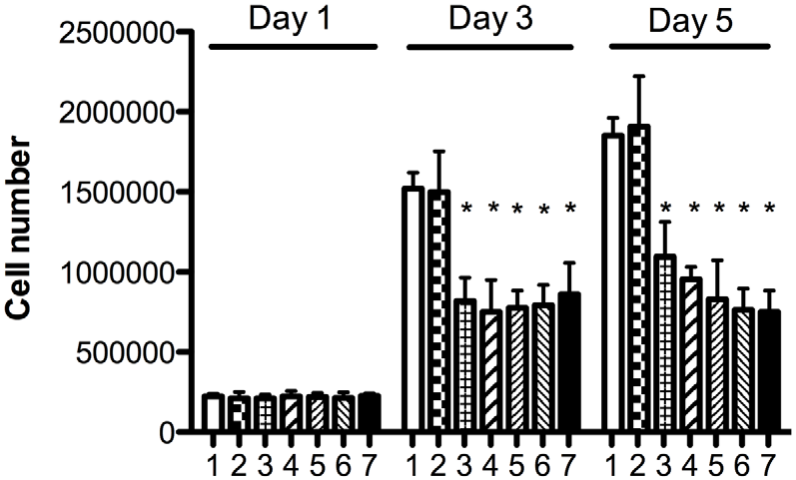
Cell counts at D1, D3, and D5 of corneal endothelial cells (CECs) grown in DMEM + F12 basal medium and seven different supplement combinations as detailed in Table 1. 1: Complete SHEM; 2: complete SHEM with heparin; 3: 5% platelet releasate (PR) with heparin, without DMSO; 4: 5% heat-treated PR (HPR) with heparin, without DMSO; 5: 5% HPR without heparin or DMSO; 6: 5% PR with heparin and DMSO; and 7: 5% HPR, with heparin and DMSO. *p<0.05 vs. condition 1.

**Figure 4:**
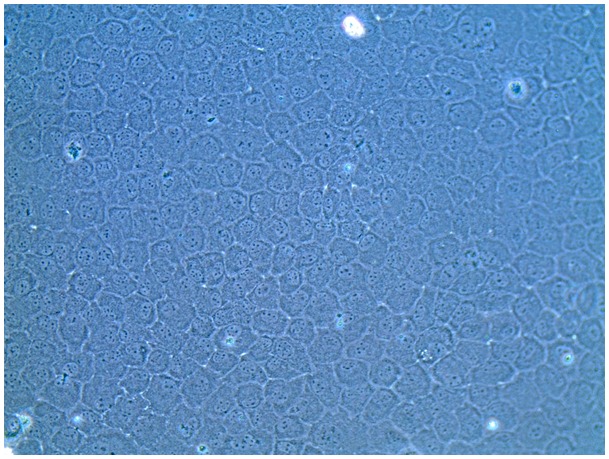
Micrograph showing the hexagonal morphology of a confluent layer of corneal endothelial cells (CECs) grown in DMEM + F12 medium supplemented with 5% heat-treated platelet releasate (HPR; magnification 200x).

The possibility of expanding CECs in basal medium supplemented with seven different combinations of supplements (as described in Table 1) containing either 5% FBS or 5% PRs as the main protein nutrient source was evaluated in 24-well plates. Growth medium was changed on days 0, 2, and 4. Cells took 5 days to reach confluence when grown in complete SHEM. Cell counts at D1, D3, and D5 are shown in Fig. 3. CECs could expand in all conditions, but significantly higher (*p*<0.05) cell growth was observed in complete medium supplemented with 5% FBS and other supplements compared to 5% PR or HPR only. MTT data on D5 confirmed that CECs exhibited good viability in the PR and HPR (data not shown). No growth medium gelation was observed in the absence of heparin, indicating that its addition was not necessary. There was no significant difference when cells were grown in PR or HPR with or without DMSO. Therefore no heparin or DMSO was used in further experiments. Optical microscopy revealed that CECs in the presence of 5% FBS, PR, or HPR grew as a monolayer with a typical hexagonal structure. However, CECs grown in basal medium supplemented with 5% HPR exhibited a more-regular shape and more-hexagonal structure than when grown with 5% PR (Fig. 4). HPR supplementation was therefore used in further experiments.

### Impact of 2.5%∼10% HPR Supplementation on CEC Growth

**Figure 5:**
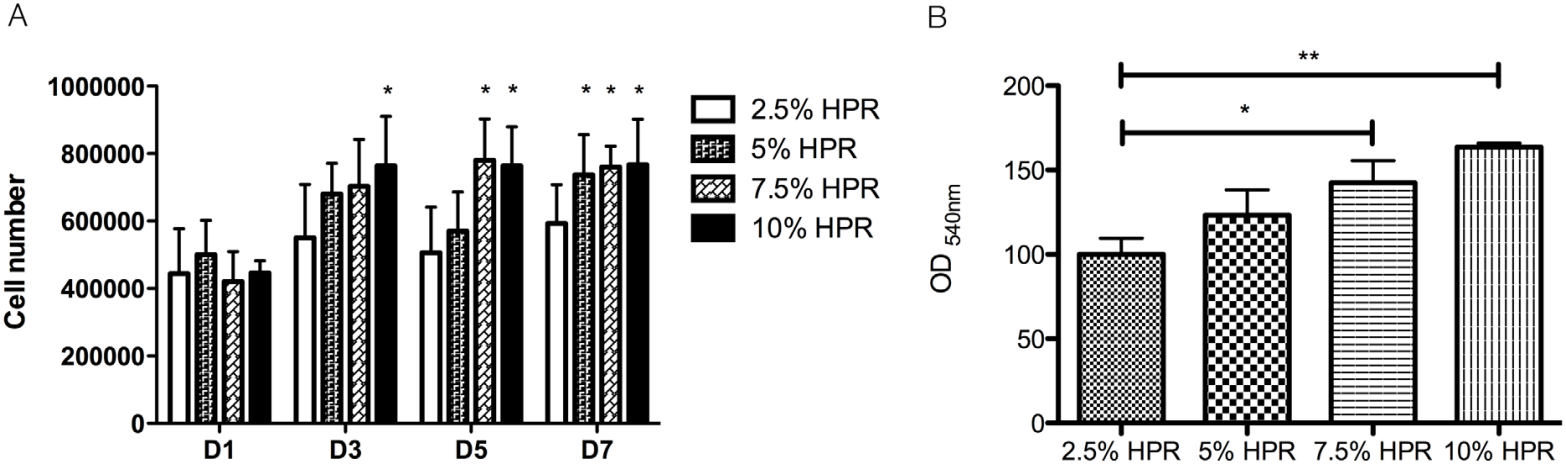
Cell count and viability assay. (A) Count of corneal endothelial cells (CECs) cultured in DMEM + F12 basal medium supplemented with 2.5%, 5%, 7.5%, or 10% heat-treated platelet releasate (HPR) for 1, 3, 5, and 7 days; (B) MTT assay of CECs cultured in DMEM + F12 basal medium supplemented with 2.5%, 5%, 7.5%, and 10% HPR for 7 days. *p<0.05; **p<0.01 compared to 2.5% HPR.

The possibility of enhancing CEC expansion by varying the content of HPR supplemented at 2.5%∼10% was evaluated over 7 days of culture. There was significant dose-dependent enhanced growth (*p*>0.05) with an increasing concentration of HPR up to 10%, compared to 2.5% HPR. Cells reached confluence at days 3, 5, and 7 when grown with 10%, 7.5%, and 5% HPR, respectively (Fig. 5A). The MTT viability assay performed on day 7 showed significantly better cell viability in medium supplemented with HPR at 7.5% (*p*<0.05) or 10% (*p*<0.01) compared to 2.5% (Fig. 5B). Therefore 10% HPR was used as the supplement for further isolation of primary CECs.

### Immunofluorescence and Western Blot Analysis of Membrane Markers

**Figure 6:**
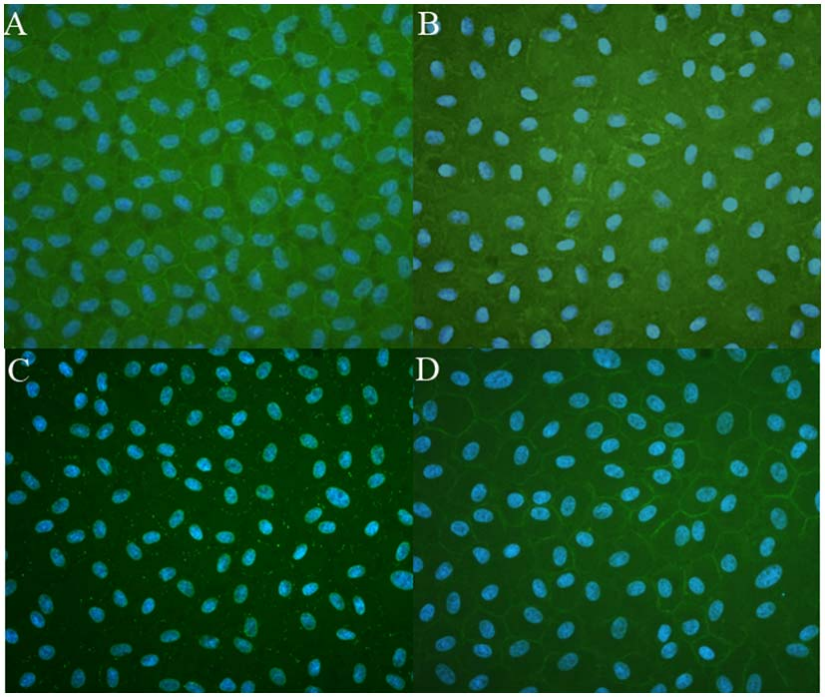
Photomicrographs of immunostaining showing expression of the membrane markers ZO-1 A), Na^+^K^+^/ATPase (B), connexin 43 (C), and N-cadherin (D) immunostaining of a confluent layer of corneal endothelial cells (CECs) isolated and expanded in DMEM + F12 medium supplemented with 10% heat-treated platelet releasate (HPR). (Cell nuclear markers were stained with DAPI. Proteins were clearly localized at plasma membranes of cells with a hexagonal shape (magnification 200x).

**Figure 7:**
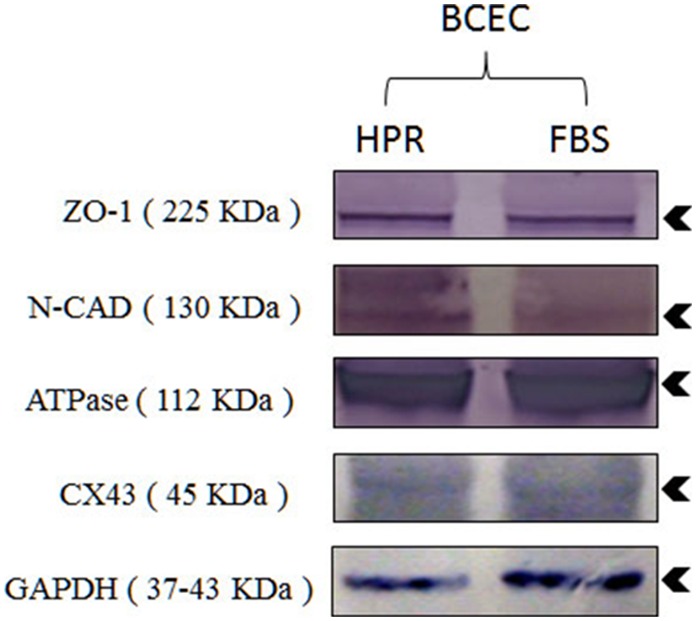
Western blot analysis of membrane markers ZO-1, N-cadherin (N-CAD), Na-K ATPase (ATPase), connexin 43 (CX43), in cells isolated and expanded in DMEM + F12 medium supplemented with 10% heat-treated platelet releasate (HPR) or complete SHEM medium (FBS). Molecular weight (kDa) of membrane markers is shown in parenthesis. GAPDH: Glyceraldehyde 3-phosphate dehydrogenase experimental marker control.

Immunofluorescent staining (Fig. 6) showed that cells isolated and expanded in basal medium supplemented with 10% HPR expressed Zo-1 (A), Na-K ATPase (B), connexin 43 (C), and N-cadherin (D). Similar levels of membrane marker expressions were obtained with cells grown in complete SHEM (not shown). A Western blot analysis (Fig. 7) showed similar expression levels for these membrane markers [ZO-1 (A), Na-K ATPase (B), connexin 43 (C), and N-cadherin (D)] in cells isolated and propagated in control SHEM or HPR.

## Discussion

Procedures for *ex* *vivo* clinical-grade expansion of CECs need to be developed to increase the supply of corneal tissues for transplants. However, the low proliferative capacity of CECs makes *ex* *vivo* expansion a challenging task. So far, successful expansion of CECs has required supplementation of growth media with complex mixtures of FBS, various other xenogenic compounds, and recombinant growth factors that are incompatible with clinical use [12]. Alternative xeno-free supplements are needed to meet requirements for clinical applications of CECs for transplantation. Our goal here was to explore the possibility of using human PRs as the sole supplement for expanding CECs. Based on our previous experience [24], we selected a PR obtained by calcium chloride activation rather than a lysate obtained by freeze-thaw cycles [28]. Indeed, this allowed us to (a) preserve the numerous protein nutrients, such as albumin (an important carrier for lipids, vitamins and hormones) transferrin or haptoglobin, present in plasma [27] and (b) remove undesirable proteins like fibrinogen that is prone to conversion into insoluble fibrin which impairs cell growth [29]. Fibrinogen removal by CaCl_2_ activation of the platelet concentrate further avoided the need for heparin which has the drawback of being of porcine origin and potentially impairs cell proliferation, at concentrations of >0.6 IU/mL [30]. From previous research, we knew that 23 mM CaCl_2_, by inducing endogenous thrombin formation, triggers platelet activation and growth factor release into the releasate [24]. Our work first identified that 5% PR could be used to expand CECs, even though cell growth was significantly less than when using full SHEM. We then observed that CECs expanded in a PR subjected to heat-treatment exhibited more-typical hexagonal morphology and good transparency. We suspect that the favorable effect of the heat-treatment was due to inactivation of the plasma complement. Recently, another HPR, developed for treating dry-eye syndrome, increased the proliferation and migration potential of ocular surface cells better than a non-heat-treated equivalent [31]. Escalating dose experiments established that significantly improved cell growth and viability were obtained by increasing HPR supplementation to 10%. In this situation, CECs still maintained a typical hexagonal shape. CECs did not undergo fibroblastic transformation [14], [32], [33] because when this phenomenon happens CECs lose their functions, which was not the case here. We verified that expressions of important cell membrane markers, in particular ZO-1 and Na-K ATPase, were seen at confluence when CECs were isolated and expanded in serum-free, xeno-free medium supplemented with the HPR. The activity of Na-K ATPase is important for the proper activity of the fluid pump and for controlling the transparency of the cornea [34], while ZO-1 ensures the tightness of cell junctions [35]. Therefore, our results demonstrated that CEC isolation and *ex* *vivo* expansion in xeno-free medium supplemented with 10% heat-treated human PR was achievable under conditions that maintain cell transparency, the hexagonal morphology, and important membrane marker expressions.

The fact that platelet materials can be used as a sole supplement to expand CECs *ex* *vivo* is very intriguing. PRs were already proven to be excellent for *ex* *vivo* expansion of human primary cells [27] including adipose-derived stem cells [36], and they have great potential in regenerative medicine and cell therapy [22], [37]. PRs provide plasma proteins and nutrients, such as sodium, potassium, magnesium, iron, and glucose. Most importantly, we showed that they contain a unique physiological mix of natural growth factors that may be crucial for CEC expansion. Studies showed the role of growth factors in the regeneration and transparency of the cornea [15], [38]. When initiating our work, we hypothesized that considering the neural crest origin of PDGF, BDNF, and VEGF neurotrophins, that are essential for brain tissues and neuronal survival, these cells may stimulate CEC growth *ex* *vivo* [2], [14]. Growth factors are known to exert important physiological roles in maintenance of the cornea [39]. PDGF-AB is present at approximately 35 ng/ml in PR. PDGF-BB promotes rat CEC growth and viability [40], whereas natural PDGF (a mixture of the AA, AB, and BB isomers), at 15∼62 ng/ml, significantly enhanced CEC growth and would healing *in* *vitro* [41]. BDNF, at around 35 ng/ml, may also be a key factor in CEC expansion. It is present in its mature 13-kDa form in PRs [26], [42]–[44]. The tropomyosin-related kinase B (TrkB) receptor, which mediates BDNF activity, is expressed in human and rabbit corneal epithelium and stroma [39], but to our knowledge, has not yet been identified in CECs. BDNF influences the development of the eye, and at 200 ng/mL, enhances the proliferation of CECs [39]. Its role in supporting CEC expansion and morphology is consistent with supplementation of growth medium with 20 ng/ml of nerve growth factor [15], [45], a neurotrophin close to BDNF [46], and by 100 µg/ml of pituitary extract, which contains BDNF [47], to support the proliferative capacity of human CECs. VEGF is present in the platelet releasates at 0.2∼0.3 ng/ml. It is also used as a supplement of CEC growth medium [13], [48]. The roles of the other platelet growth factors cannot be excluded. For instance, EGF, which was present at approximately 0.45 ng/ml in our PRs is commonly used at 2∼5 ng/ml to supplement CEC expansion media [12]–[14], [49], but 0.01 ng/ml was found sufficient to promote the growth of rabbit CECs [15]. EGF receptors are expressed by CECs [50]. EGF promotes the proliferation of human CECs in vitro [49] and in vivo, improves eye tissue hydration by stimulating fluid transport in the corneal endothelium [51]. b-FGF, which promotes mitosis of CECs, is sometimes used, at 2∼10 ng/ml, in CEC expansion media [48], [52], [53]. It was present in our PRs only at 0.1∼0.5 ng/ml, a dose which might be sufficient to enhance CEC growth when combined with the other platelet growth factors. The role of TGF-β may be more ambiguous as it may at certain concentrations alter CEC expansion and induce formation of myofibroblasts and corneal scarring [38], [54] and lead to endothelial-mesenchymal transition [33]. Conversely, TGF-β exerts an anti-inflammatory role in ocular disorders and infections [15]. Testing a platelet lysate depleted in TGF-β through adsorption on an anion-exchanger [55] may be of interest. HGF, which was present at about 0.3 ng/ml in our PRs, is known to stimulate proliferation of CECs in a dose-responsive manner, consistent with the presence of the HGF receptor on the cell surface (MET) [56], [57]. Bovine insulin and insulin-like growth factor (IGF)-1 are also used to supplement CEC culture media [48], [53], [58]. Although we did not measure IGF-1, which is structurally homologous to insulin [59], in the PR or HPR, our previous studies demonstrated that it is present in the plasma compartment of PRs at approximately 50∼70 ng/ml [24], so we believe that it was present at similar concentrations in the PR and HPR. Therefore, the natural mix of growth factors present in platelet releasates may be a key element for the isolation and expansion of CECs.

## Conclusions

This work establishes for the first time a proof of concept that human PR rich in growth factors can be used as a supplement for serum-free, xeno-free *ex* *vivo* expansion of CECs. These findings open attractive perspectives for clinical-grade use of CECs for transplantation in patients suffering from corneal diseases. Further work is needed to confirm these data using human CECs and develop a culture system on a suitable substrate for corneal grafting.
